# Structural Water Stabilizes Protein Motifs in Liquid Protein Phase: The Folding Mechanism of Short β-Sheets Coupled to Phase Transition

**DOI:** 10.3390/ijms22168595

**Published:** 2021-08-10

**Authors:** Dóra Papp, Imola Csilla Szigyártó, Bengt Nordén, András Perczel, Tamás Beke-Somfai

**Affiliations:** 1 Laboratory of Structural Chemistry and Biology, MTA ELTE Protein Modelling Research Group, Institute of Chemistry, Eötvös Loránd University, Pázmány Péter Sétány 1/A, H-1117 Budapest, Hungary; dorapapp@chem.u-szeged.hu (D.P.); perczel@chem.elte.hu (A.P.); 2 MTA-SZTE Lendület Computational Reaction Dynamics Research Group, Interdisciplinary Excellence Centre, Department of Physical Chemistry and Materials Science, Institute of Chemistry, University of Szeged, Rerrich Béla tér 1, H-6720 Szeged, Hungary; 3 Biomolecular Self-Assembly Research Group, Institute of Materials and Environmental Chemistry, Research Centre for Natural Sciences, Magyar tudósok körútja 2, H-1117 Budapest, Hungary; szigyarto.imola.csilla@ttk.hu; 4 Department of Chemical and Biological Engineering, Physical Chemistry, Chalmers University of Technology, SE-412 96 Göteborg, Sweden; norden@chalmers.se

**Keywords:** membraneless organelles, quantum mechanics, liquid–liquid phase separation, protein folding/unfolding

## Abstract

Macromolecular associates, such as membraneless organelles or lipid-protein assemblies, provide a hydrophobic environment, i.e., a liquid protein phase (LP), where folding preferences can be drastically altered. LP as well as the associated phase change from water (W) is an intriguing phenomenon related to numerous biological processes and also possesses potential in nanotechnological applications. However, the energetic effects of a hydrophobic yet water-containing environment on protein folding are poorly understood. Here, we focus on small β-sheets, the key motifs of proteins, undergoing structural changes in liquid–liquid phase separation (LLPS) and also model the mechanism of energy-coupled unfolding, e.g., in proteases, during W → LP transition. Due to the importance of the accurate description for hydrogen bonding patterns, the employed models were studied by using quantum mechanical calculations. The results demonstrate that unfolding is energetically less favored in LP by ~0.3–0.5 kcal·mol^−1^ per residue in which the difference further increased by the presence of explicit structural water molecules, where the folded state was preferred by ~1.2–2.3 kcal·mol^−1^ per residue relative to that in W. Energetics at the LP/W interfaces was also addressed by theoretical isodesmic reactions. While the models predict folded state preference in LP, the unfolding from LP to W renders the process highly favorable since the unfolded end state has >1 kcal·mol^−1^ per residue excess stabilization.

## 1. Introduction

The environment effects on protein structure have recently experienced an increased interest due to its importance in diverse areas, from biofilms through chaperones to membraneless organelles [1,2]. The latter are micrometer-sized protein-rich droplets in which a liquid–liquid phase separation (LLPS) takes place and exhibits vastly different properties when compared to water regarding their magnitude of dielectric constant, viscosity, ionic strength and hydrophobicity. Similar local environment effects may occur in the internal region of bulky protein complexes, such as the GroEL/GroES [3] or ClpXP [4] systems. These assemblies provide examples of a uniquely important area where biophysical and biochemical processes take place [5], which can involve very different, even opposite, directions of reaction, i.e., protein folding and unfolding. Characteristic cellular organelles with proteins as major components [1,6] have multiple short motifs which are initially disordered, but upon undergoing LLPS they often form short β-sheet rich domains, or LARKS (low-complexity aromatic-rich kinked segments) [2,7]. Related to this, the self-assembly of similar short-motif peptides has been studied for phase transitions from solution into crystals or fibrils [8,9] and also for the reverse process of fibril disassembly [10].

However, for the desirable future control of the folding and unfolding criteria in liquid protein (LP) systems, a better understanding of the thermodynamic and molecular-level mechanistic details in these processes would be key. The energetics of such systems is complex, as both enthalpic and entropic terms are influenced by the bulk environment, peptide conformational changes, peptide–peptide interactions, peptide-water H-bond formation, partial desolvation and also the appearance of structural water in folded regions. The change in the environment hydrophobicity can result in a change of buried H-bonds strength as demonstrated, e.g., for DNA base pairs [11], and these effects often seem to be associated with enthalpy–entropy compensation [12]. For amyloids, a significant recent progress in determining kinetic and thermodynamic properties is achieved for short motif systems [13,14]. However, the overall folding contributions and preferences of the peptide backbone are often concealed by the dominance of the particular side chains of the studied sequence. In experiments performed on dipeptide assemblies, the Gibbs free energy can be in a range of −1.76 < Δ*G* < −0.12 kcal·mol^−1^ per residue depending on the side chain composition [13,14]. In contrast to amyloids, the formation of membraneless organelles is even more complex, as a significant amount of water is retained in the LP phases [1]. Therefore, to obtain further insight on the effect of phase separation, as well as phase transition for the unfolding-folding processes, we focus here on the mechanism and the energetic properties of small parallel and antiparallel β-pleated sheet motifs in the presence of ‘lubricating’ waters.

Previous results on these motifs helped in understanding their stability and dynamics during folding or structure disrupting events [15,16,17,18]. Molecular dynamics (MD) simulations, together with single-molecule force microscopy, studied the formation and break-up of H-bonds as a function of the pulling direction [19] and the composition of the solution matrix [20]. The picture is complex as there are several challenging problems for quantitative atomic level description, including the limited accuracy of force fields for describing H-bonded systems [21,22]. However, due to their structural degree of freedom, β-sheet motifs are suitable for QM calculations [23,24,25]. These calculations are increasingly observed in such studies as they can provide an accurate description of the energetics during a step-by-step investigation of dynamic processes and can also readily employ implicit solvent models with diverse bulk conditions [26,27,28].

As previously reported [23,25], the unfolding of short β-sheet motifs by QM calculations, demonstrated that both structural and bulk water molecules are required to reach a complete description, that matches experimental results. Due to its relative simplicity, the modeling of separating β-sheet motifs is an established method for obtaining valuable insight into mechanical aspects of protein unfolding and also for the reverse process analogous to protein single molecule force experiments [29,30] or chemically induced unfolding [14,31]. By varying the surrounding bulk conditions and by employing the isodesmic reaction approach, here we investigate how folding properties of the employed model systems are affected when passing from W → LP phase and vice versa. The results indicate how the delicate balance of folding stability is altered by the LP phase and how, upon phase separation, the W → LP or the LP → W transitions change in a manner that may be exploited by natural systems in order to optimize energy requirements of folding-related and unfolding-related events. Accordingly, kinetic barriers are shown to be highly dependent on the hydrophobicity of the environment, the latter being more favorable for folding into a secondary structure. The QM calculations also demonstrate that the sensitivity of the system to bulk changes is greatly enhanced by the presence of structural water molecules.

## 2. Results and Discussions

### 2.1. The Energy Profile of Lateral Unfolding in W and LP Environments

**Figure 1:**
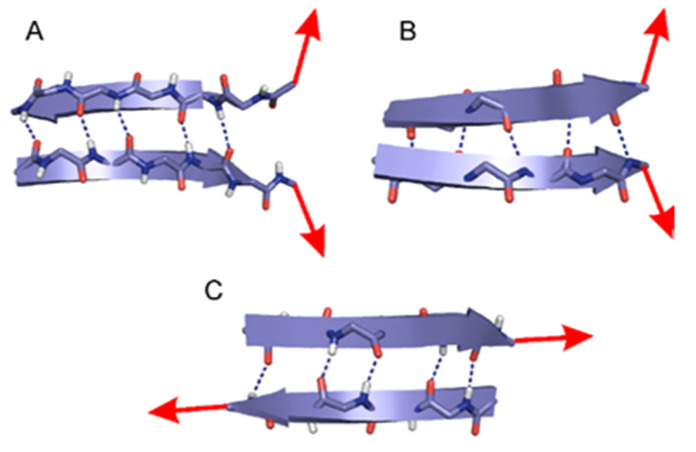
Schematic description of lateral and sheared unfolding directions of β-sheet motifs studied here. Secondary structures shown as ribbons and backbone atoms as sticks. Interstrand H-bonds indicated by dark blue dashed lines. (**A**) Lateral unfolding of antiparallel β-sheet (AP) motif. (**B**) Lateral unfolding of parallel (P) β-sheet motif. (**C**) Sheared unfolding of an AP motif.

Prior to understanding the relation between sheet stability and phase separation, several models and QM methods had to be fine-tuned, enabling comparison in different matrices. Accordingly, both parallel and antiparallel β-sheet structures were subjected to DFT calculations using the B3LYP functional (Figure 1 and Figure S1). B3LYP is perhaps the most popular density functional in use, even though its limitations have been recently spelled out [32]. When considering properties of small peptidic and H-bonded systems, its accuracy and its low computation cost still renders it a reasonably reliable approach, with energy values falling in the chemical range expected [33,34,35,36,37]. This is especially the case when one uses triple-ζ basis sets, which also include polarization and diffuse functions where experimental results can be well reproduced or predicted for conformation-sensitive H-bonded peptidic systems [38,39]. 

Our previous results [23] regarding the mechanism of lateral unfolding showed that, in parallel β-sheet models, the water molecules keep moving toward the interior of the sheet where the remaining H-bonds are still connecting the two strands. These water molecules form H-bonds with the bridge atoms of peptide–peptide H-bonds that are about to break up, aiding their separation (Figure S2). For antiparallel models, this type of ‘water-walking’ can be observed only when a sufficient number of water molecules are present, i.e., for the model with three H_2_O molecules [23].

**Figure 2:**
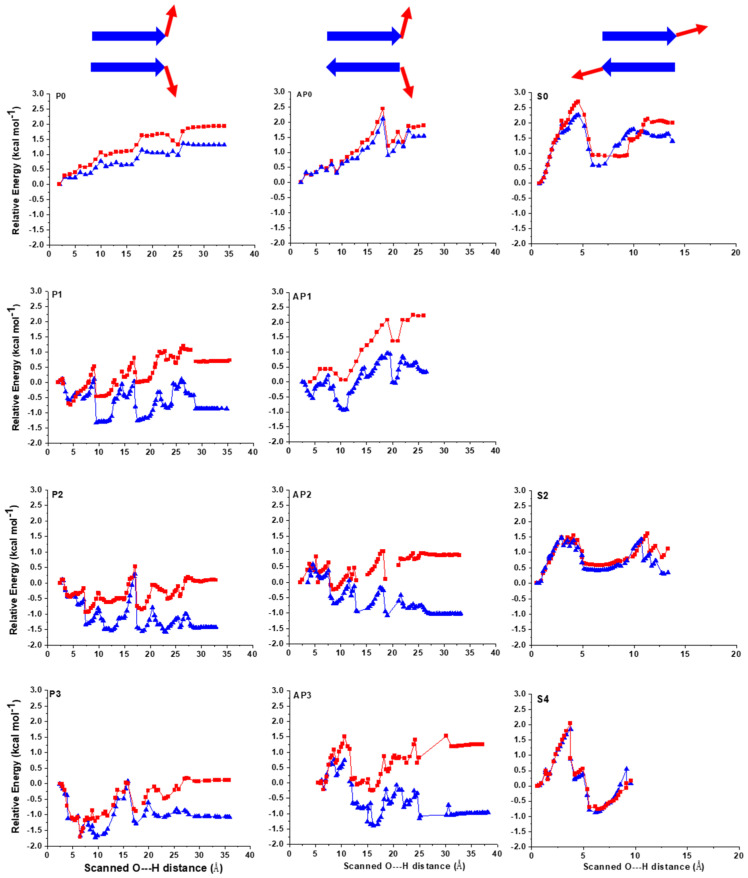
Lateral and sheared unfolding of parallel and antiparallel β-sheet models. TOP: models with 0 water molecules for lateral parallel (P0), lateral antiparallel (AP0) and sheared unfold (S0). MIDDLE: models with 1 or 2 water molecules for lateral parallel (P1;P2), lateral antiparallel (AP1;AP2) and sheared unfold (S2). BOTTOM: Models with 3–4 water molecules for lateral parallel (P3), lateral antiparallel (AP3) and sheared unfold (S4). Relative energies are in kcal·mol^−1^ and normalized to the number of amino acids present in the models. LP environment is shown as red; W as blue line.

Here, the relative energy of these dimer β-sheet models was investigated both in W and in LP phases (Figure 2). The relative energy values show significant differences depending on the local environment. Considering a polar aqueous media during unfolding, the relative energies decrease throughout the process for most of the models (Figure 2).

Regarding the lateral unfolding of the models with explicit water molecules, in each step when a peptide–peptide H-bond is broken up or a new peptide-water H-bond is formed, the Δ*E* curve experiences a rapid change. The break-up events of the initial peptide–peptide H-bonds are associated with rapid drops in energy for both W and LP environments coupled to the entering of the water molecules between the peptide strands, stabilizing the structure [23] (Figure S2 and Figure 2). The break-up of inter-strand H-bonds is more favorable in W compared to LP because of the solvation of the polar groups previously involved in the inter-strand H-bonds. In the case of W, the initial rapid decrease in energy is mostly followed by a slight further stabilization as additional inter-strand hydrogen bonds are broken up; thus, for most of the models, the unfolded end states in W remain energetically lower than the initial folded motifs (Figure 2). This is not surprising, since initially the β-strands are not as exposed to the surrounding media while they gradually become exposed during separation. These results are in full agreement with experimental studies, namely that most short peptide sequences will readily dissolve in W. By contrast, in the LP environment, the solvation of the polar groups is less favored due to the low surrounding relative permittivity. For the LP phase these will result in higher or approximately equivalent relative energies to those of the initial structures (for more details see Methods and SI).

When considering the relative stability of the separated end states, the final averaged difference in energy values between the two environments are ~9 kcal·mol^−1^ for the parallel motifs and ~13 kcal·mol^−1^ for the antiparallel ones, which is ~1.5 kcal·mol^−1^ and ~2.2 kcal·mol^−1^ per amino acid, respectively. These values are significant considering the small size of the models. Firstly, this demonstrates that the nature of the solvent influences the relative stability of both types of β-sheet structures similarly, with W being more favored. Secondly, it also suggests that the antiparallel β-sheets are more stable than the parallel ones, which is in line with previous studies [40].

### 2.2. Sheared Unfolding in Different Surrounding Media

The effect of sheared or longitudinal unfolding on peptide β-sheet motifs was also studied in the presence of structural water molecules (Figure 1 and Figure 2) for both W and LP. In this case, the interaction of water molecules with the peptide strands was expected to be different compared to lateral unfolding. While for lateral unfolding, the water molecules enter between the peptide strands from one terminal; here, it is more probable that several water molecules can simultaneously enter between the strands as more inter-strand H-bonds are affected (Figure S1). In the sheared unfolding process, the inter-strand peptide H-bonds do not break up in a stepwise manner for the models with structural waters, but they break up rather simultaneously, which is in line with previous experiments [30] and MD simulations [19,20]. However, the presence of explicit structural waters and the application of QM optimization reveals that the abrupt break up of H-bonds does not immediately result in complete β-sheet separation. The explicit water molecules that were initially coordinated to the two opposing O-bridge atoms of the two peptides [20,40] move in between the two strands, making a single layer of water molecules between them and holding both peptides by H-bonds. This, in principle, results in a breakup of the initial H-bonds, as the water molecules between the strands form a ‘buffer’ for maintaining the original peptide–peptide interaction. The special coordination of the initial water molecule on the inter-strand H-bonds allows the formation of several bifurcated ones. Though these newly formed peptide-water H-bonds can be less favorable than the original ones, they still keep the peptide strands in close proximity, allowing for the fold to be restored.

The energetics of the sheared unfolding direction supports the mechanism described above. While the initial peptide–peptide H-bonds are continuously replaced by peptide–water H-bonds with the first layer of water molecules, the relative energy of the system increases very rapidly (Figure 2). Once the first peptide–water–peptide H-bond pattern breaks up, the relative energy quickly drops and the two peptide strands shift one register of peptide bonds relative to each other. An interesting energetic difference compared to lateral unfolding is that the solvent effects here are much less pronounced (Figure 2). The relative energy differences between LP and W are also nearly insignificant for all models. The main reason for this drastic difference compared to lateral unfolding is that, for sheared unfolding, the water molecules entering between the strands ‘shield’ the peptide strands from an apolar environment, and thus its negative energetic effect is attenuated. During lateral unfolding, the polar regions are increasingly exposed to the surrounding media, resulting in an additional 6–7 polar groups in direct interaction with the bulk environment compared to the initial state. By contrast, for the sheared unfolding only 2–3 additional polar groups are in direct connection with the bulk surrounding by the end of the simulations.

### 2.3. Larger Models—Consecutive Lateral and Sheared Unfolding Results in Strand Shortening

**Figure 3:**
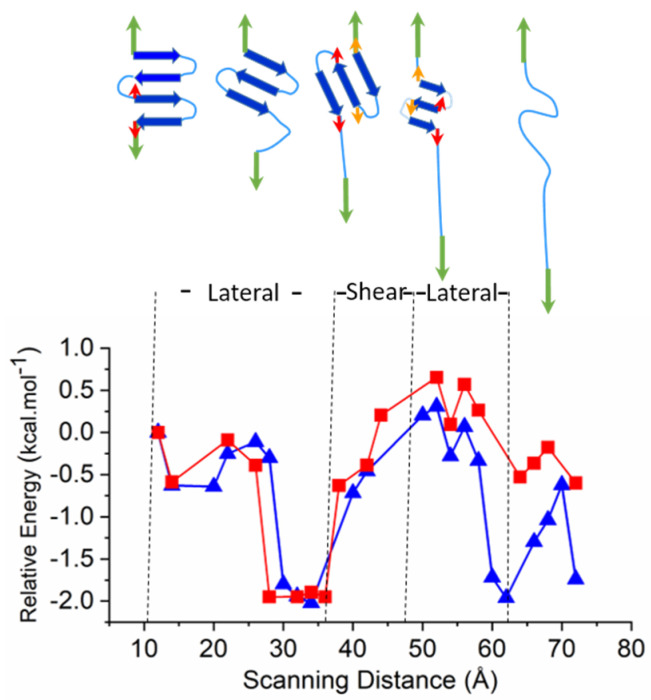
Unfolding of the 3Turn model and the associated normalized relative energy as a function of scanning distance. Relative energy (kcal·mol^−1^) normalized to the number of residues participating in the unfolding process, either lateral or sheared (longitudinal). LP environment is shown as red; W as blue.

In order to compare changes in mechanism and relative energies, a larger β-sheet model (3Turn) was also investigated. This model consists of a total of 22 residues and its unfolding was tested in both W and LP environments. In order to facilitate comparison, the length of the β-strands was chosen to match the models used for sheared unfold. Similarly to the smaller models, nine water molecules are present in 3Turn which enter between the strands and mediate the process of unfolding. However, for this large model the entire unfolding process can also be separated into lateral- and sheared unfolding periods. The initial phase consists of the lateral unfolding of the last strand on the N-terminal (Figure 3). Once the unfolded N-terminal is stretched, the relative position of the two pulling directions becomes such that the process changes to sheared unfolding, with all remaining β-strands involved. This results in a rapid increase in energy, and the ‘melting’ of the structure with the inter-strand H-bonds is shifted, and the sheared process finally results in a shorter β-sheet (Figure 3 and Figure S3). Next, the unfolding mechanism switches back to lateral and involves all strands. Finally, the sheet motif collapses into mainly stretched regions, where the individual amino acids first adopt turn-like γ_L_ [41] conformations on the Ramachandran Map; finally, they are stretched into extended β_L_ conformations.

The energy profile during the unfolding of the 3Turn model supports the separation of the entire event into lateral and sheared unfolding sections (Figure 3). The initial lateral unfolding of the N-terminal fourth strand (Figure 3, Figure S3 and Video SI_3Turn_9w_close) is followed by a rapid increase in energy at ~36 Å scanned distance, which is associated with the sheared unfolding acting on all the three remaining strands. By the end of this period, at ~46 Å, the sheared unfold results in a partially melted and shorter three-stranded β-sheet, where one residue is ‘pulled out’ at both of the terminal sheets. Finally, a lateral unfold takes place, and the three-stranded system unfolds simultaneously.

Regarding the two different environments, similarly to the shorter models, the unfolding in water is energetically much more favored compared to that in LP. By contrast to shorter motifs, the energy difference here starts to be observable after the major shear event as an increasingly larger part of the polypeptide backbone becomes exposed to the bulk environment (Figure 3).

### 2.4. Energetic Aspects of Coupling Phase Changes to Folding/Unfolding Events

**Figure 4:**
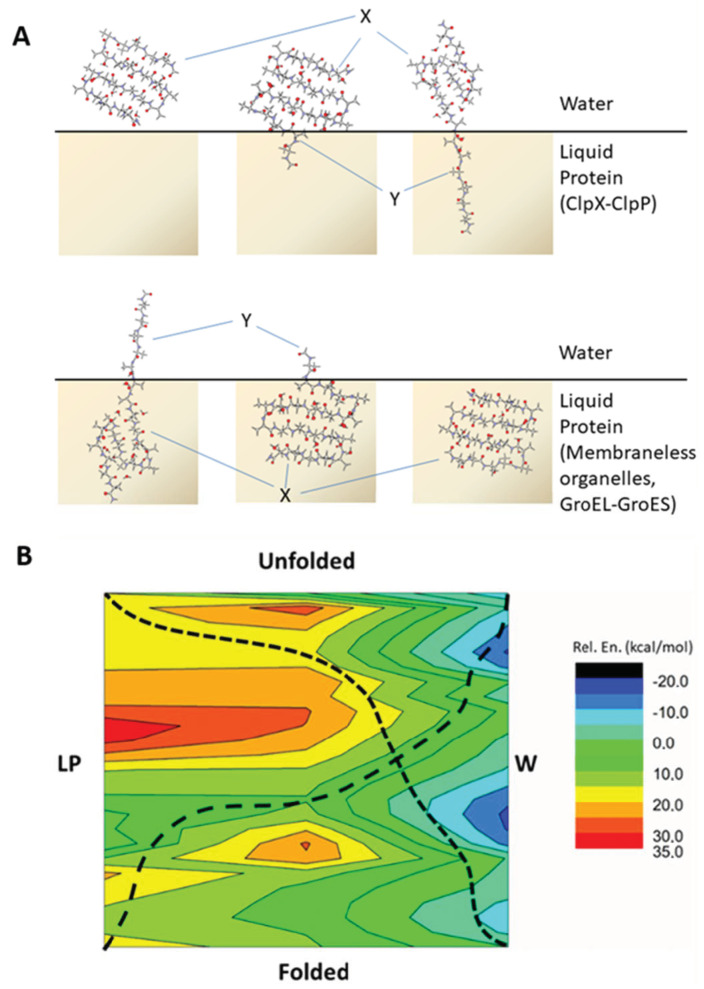
(**A**) Schematic description of protein motif unfolding coupled to changing bulk environment. In order to obtain the relative energy of each motif, E(X-Y), a splitting strategy was employed together with isodesmic reactions according to Equation (S3) (for details see Methods in Supplementary Material). At various stages of the unfolding process the model was split into two parts surrounded by water (X) and a bulk protein environment (Y), respectively (TOP). The same procedure can be applied to a process where the structural change is met by a reverse change of bulk environment (BOTTOM). For more details, see Materials and Methods. (**B**) Schematic relative energy surface coupling folded and unfolded states of 3Turn with phase transition. The surface is based on the investigated 3Turn models and isodesmic reactions as detailed in text. The potentially optimal reaction coordinates are displayed by dashed lines for both the Folded ↔ Unfolded (W ↔ LP) and Folded ↔ Unfolded (LP ↔ W) scenarios.

Isodesmic reactions provide a well-established method for understanding virtual chemical reactions combining energetic properties of different reactants in order to approach energies of products. This method is also appropriate for assessing conformational properties [25,42,43]. Accordingly, the relative energies during the unfolding of the 3Turn model, upon changing between W and LP, were estimated by isodesmic reactions. Amino acids were assumed to enter from one phase to the other one by one (Figure 4). We have calculated fragments of 3Turn along its unfolding split between the LP and W environments, both for LP to W and for the reverse process (for details see Methods, Figure 4 and Figure S4). The resulting sums provided the corresponding relative energy diagrams using the total energy of the folded 3Turn in W as a reference.

Firstly, we investigate the LP to W phase change coupled to unfolding, which may occur during the processes of, e.g., LLPS dissolution, during ordered peptide assemblies leaving lipid membranes or during partial unfolding in larger proteins with a more buried core. Here, we observe a relatively flattened energetic profile during the initial part of the transition. The rapid entering of the water molecules between the N-terminal peptide strands occurs while the entering of this part into W takes place. Without a phase change, the sheared unfolding presents a high barrier in both W and LP. However, upon being coupled to phase change, this barrier is significantly lowered by the elongated part of the peptide being solvated in W. By the end of the unfolding, the model experiences a rapid and large stabilization in W (Figure S4). The LP to W transition coupled to unfolding has a large stabilization on the peptide backbone, and the energy gain is close to 1.5 kcal·mol^−1^ per residue. This is much higher than when directly moving the folded model from LP to W, which can be estimated to be at ~0.9 kcal·mol^−1^ stabilization per residue. Note, that the latter value on our Ala containing models closely follows the experimental results obtained by Wimley, Creamer and White, where the Whole Residue Hydrophobicity Scales predict a + 0.5 ± 0.12 kcal·mol^−1^ change in Gibbs free energy for Ala when entering from water to n-octanol [44,45]. The coupling of phase transition to unfolding also shows a significant decrease in the barrier height observed for the sheared unfold. This part of the unfolding process results in a ~16 kcal·mol^−1^ difference in relative energy, much smaller than the ~24 kcal·mol^−1^ value observed for an unfold in the LP phase without phase change. The step-by-step monitoring of the phase transition also reveals that the large energetic differences between the initial and end states start to appear when most part of the model is unfolded and has already entered into W.

The other direction, where unfolding is coupled to W to LP phase change, is analogous to, e.g., how the enzyme unfoldase or disassembly chaperone ClpX unfolds peptide segments coupled to ATP hydrolysis, where the unfolded sequence enters to the middle of the enzyme pore (Figure 4) [46]. Note, however, that while in principle the special side-chain composition of the entering sequence does not alter chaperone function much [46], it is clear that specific side-chains may have a substantial effect at molecular level on the actual mechanism and energetics [47]. The unfolding of 3Turn along the W to LP transition is energetically disfavored throughout the entire process, with a relative energy difference of ~+0.65 kcal·mol^−1^ per residue observed (Figure 4). This value is lower than that observed for smaller models; nevertheless, in line with the ATP requirements, it still indicates that, in general, a significant amount of energy needs to be invested to make unfolding and simultaneously transfer into the LP phase feasible. However, such a combination of the two processes may still be energetically beneficial in terms of passing through the kinetic barriers associated with the sheared unfolds as the high energy barrier can seemingly be smeared with the phase transition. Accordingly, for the isodesmic model following the W to LP transition, the barrier height for the sheared unfolding part is smaller than the ones observed either simply in W or in LP unfold (Figure 4). It could be proposed that entering into the more non-polar environment and unfolding at the same time results in a monotonically increasing relative energy for the process. This may provide an optimal scenario for enzyme complexes such as the ATP-dependent Clp [48]. Note, however, that the barrier potentially related to the sheared unfold renders the energy investment more localized in the latter example, which is in line with observation on the function of ClpX where, in the case of mechanically stable proteins, several ATP consuming attempts are needed to reach progress [46].

### 2.5. Comparison to Experiments

All results presented here are based on QM calculations on simplified peptide models which describe structures and bonding properties of the complex protein–water systems at a high level of theory. Nevertheless, to obtain a more complete picture, one has to complement the predicted major components of Δ*H* values by a *T*Δ*S* term that includes conformational entropy in order to be comparable with experimental Gibbs free energy values. These are not available within the limits of the present QM calculations; nonetheless, they can be qualitatively estimated due to the details provided on related systems [13,49]. It has been shown that short chain peptides from amyloid sequences show relatively low −*T*Δ*S* of crystallization at the 290–310 K temperature range, in the order of magnitude of −0.7–−0.1 kcal·mol^−1^ per residue [13,14]. Compared to this, our relative stabilization energy values of 0.9 to 2.2 kcal·mol^−1^ per residue for antiparallel short models towards folding indicate that the enthalpic term is much more significant than the entropic one. Furthermore, the addition of monomers is on the level of 1.2 to 1.8 kcal·mol^−1^ energy gain [13,14], which correlates well with our value. Thus, it is relevant to discuss how serious a neglect of entropy really is for the current conclusions. Clearly, hydrophobic effects are very important, and the question of to what extent the large negative Δ*Hstruc* of structured water is compensated by the potentially equally large negative *T*Δ*Sstruc* usually resulting in fairly small negative Δ*Gstruc* values giving rise to the well-known entropy-enthalpy compensation has to be addressed [12]. Furthermore, Δ*Gstruc* will become smaller and smaller (less negative) with increasing temperature, and thus approaching Δ*Hstruc* = *T*Δ*Sstruc*, the characteristics of a ‘microscopic phase transition’ [12]. On the other hand, if we consider the extreme situation of a liquid solution, such as the state when the protein molecules are fully solubilized and disordered, one may argue that, for the increasingly folded and/or aggregated state, we will have a situation closely resembling a precipitated crystal where the structure and its stability can be predicted by Δ*H* values obtained by quantum mechanics. According to a recent experimental study of DNA destabilization in semi-hydrophobic media (decreasing the hydrogen bonding power of water), a reduction in water chemical potential (or solvent dielectric permittivity) results in a corresponding reduction in the hydrophobic stacking attraction behind the base-pair stacking, while a reinforcement is simultaneously noticed for the Watson–Crick hydrogen bonds holding the base-pairs together because of the diminished competing hydrogen bonding from abundant water [11]. Similarly, we can expect that the presence of hydrophobic surfaces has an effect upon protein folding that results in stronger discrete H-bonds in the structure involving structural waters, which would make Δ*Hstruc* even more negative. The negative entropy from the ordering of peptide residues may be at least partly compensated by a positive entropy contribution from released unstructured water. This method of reasoning could justify our neglect of entropy and the assumption of using the present QM results as a guide to preferred structure.

## 3. Materials and Methods

### 3.1. Quantum Mechanical Calculations

Computational studies were carried out by using the Gaussian 09 software package (Rev.A.1). All smaller models employed to describe either lateral or sheared unfolding (Figure 1) were previously optimized [23,25]. The optimized geometries, more than 1200 overall, were submitted for single-point calculations at the B3LYP/6-311++G(d,p)//B3LYP/6-31G(d) level of theory, which resulted in a higher level energy profile and renders the basis set superposition error (BSSE) negligible [25,42,50,51]. In order to address the effect of bulk environments, the integral equation formalism for polarizable continuum model (IEFPCM) [52] was employed for both the W and LP phases. The LP environment was specified by parameters commonly used for modeling a bulk protein environment with dielectric constant of 4 and by using 2.5 Å solvent radii with a united force field description (UFF) [26,27,28].

Formyl (For) and NH_2_ protecting groups were used on the peptide terminals that did not initially coordinate with the explicit water molecules. The effects of H-bonds that potentially formed due to this protection at the end of simulations were removed from the energy profiles in all simulations and were also omitted from discussion. The break-up of each H-bond could be assigned to a relatively short ~0.4 Å interval, which was also coupled to an abrupt large increase in the distance of the involved (C=)O---H(-N) bridge atoms. This usually took place in a one scan step, and then the distance between the two O---H atoms increased above 2.5 Å.

### 3.2. Employed Models

#### 3.2.1. Lateral Unfolding

Two-stranded peptide models with both parallel (P) and antiparallel (AP) β-sheet structures were optimized at the B3LYP/6-31G(d) level of theory. These models were investigated in vacuum (P0 and AP0) as well as with one, two and three water molecules P1, AP1, P2, AP2 and P3 AP3, respectively, with the waters H-bonded to one another and coordinated to the terminal part of the models. (Figure S1) The P0, AP0, AP1 and AP2 models consisted of two peptide strands [For-(Ala)_3_-NH_2_]_2_ while, in order to avoid unwanted hydrogen bonds, the models P1, P2 and P3 had an acetyl protecting group (Ac) at the N-terminal, [Ac-(Ala)_3_-NH_2_]_2_. For AP3, one strand was protected at the N-terminal Ac-(Ala)_3_-NH_2_ and the other at the C-terminal For-(Ala)_3_-NHMe. In all models the water molecules were initially coordinated on one side of the terminal H-bond to mimic the coordination of the first water molecules approaching to an otherwise buried sheet motif. These optimized structures were used as the starting geometry for a relaxed potential energy surface scan along the distance (i.e., scanned distance) of the terminal C=O---H(-N) hydrogen bond. The stepwise progression of these scans was 0.2 Å and was performed until all the interstrand H-bonds broke up between the two peptide strands, and they became fully separated (~35 Å scanned distance). In the case of the sheet models with three water molecules, convergence was considerably longer; therefore, the scan was performed with 0.4 Å step size above 9.0 Å distance.

#### 3.2.2. Sheared Unfolding

Two-stranded AP sheet peptide models were employed and submitted for optimization at the B3LYP/6-31G(d) level of theory. As these models investigate the sheared unfolding mechanism which is rather different from the lateral one, both the employed sheet models and the distribution of water molecules accompanying the sheet structures were chosen altered. The models consisted of two longer peptide strands [For-(Ala)_4_-NH_2_]_2_ and, thus, had initially 5 interstrand H-bonds. In contrast to lateral unfolding mechanism where the water molecules can enter between the neighboring strands from one terminal of the sheet motif, the structural water molecules are likely to play a role along the entire sheet structure for sheared unfolding. Consequently, in addition to the model in vacuum (S0), models with two and four water molecules, S2 and S4, were also employed, which had their water molecules coordinated on two carbonyl oxygens along the entire motif (Figure S1C). In the case of the S4 model, the additional two water molecules were coordinated on the first waters. Note that, the longitudinal unfold of parallel motifs is rather unlikely during protein unfolding; thus, it was not considered (Figure 1).

#### 3.2.3. Larger Models

In order to investigate the two different mechanisms combined together, a more extended β-sheet model of 22 alanine residues with a total of 254 atoms containing 3 turn regions and four β-strands was also prepared (3Turn). A total of 9 water molecules, representing structural water, were positioned into the ‘clefts’ of the sheet and was optimized initially by using the semi-empirical PM6 method (Figure S5), which was developed for investigating biochemical systems and was found superior in this regard to previous methods [53,54]. 3Turn was then submitted to a stepwise unfolding, where the distance of C-atoms and N-atoms of the two terminal carbonyl and amino groups was scanned with 0.2 Å steps at the PM6 level of theory. In order to provide more accurate structure for higher point energy calculations, selected structures taken at every 2 Å along the scanned distance were submitted for further optimization at the B3LYP/3-21G level of theory. Finally, single point calculations were performed on these optimized models at the B3LYP/6-31+G(d,p) level of theory by using IEFPCM continuum solvent models mimicking both W and LP environment as described above.

The energetic effect of phase separation is detailed in Supplementary Materials.

## 4. Conclusions

Based on the QM calculations presented, it is clear that aqueous and liquid–protein environments have a strong effect on the folding properties and affinity of protein and peptide segments. The present investigation provides insight at the atomic-level to structural and energetic properties of the mechanical unfolding of small peptide models under the influence of two bulk environments: the aqueous (W) and a membrane-like liquid protein phase (LP) (Figure 4A). Our results demonstrate that the structural water molecules present on the studied peptide motifs are crucial in aiding unfolding, keeping partially unfolded peptide strands connected via H-bonds formed between bridge water molecules and the peptide backbone; however, they also render the folding/unfolding energetics sensitive to molecular environments. The water-aided lateral unfolding of both parallel and antiparallel β-sheet models showed that the unfolding event is favored in W, but it is highly disfavored in an LP-like molecular environment. The calculations indicate that the average stabilization in W relative to LP can be as high as ~2.2 kcal·mol^−1^ per amino acid in case of the unfolding of antiparallel motifs. This significant value also explains why disordered sequences have high tendency to become folded in environments similar to the LP phase that is in lipid bilayers, in protein cores or in the liquid protein phase of membraneless organelles. In contrast to lateral unfolding, the longitudinal or sheared unfolding direction induces much higher energetic barriers for the process, but a less sensitive environment dependency. These conclusions were fully supported by following the unfolding of a larger model, where both lateral and sheared directions are present (for clarity, see Video S1 in Supplementary Material). Isodesmic reactions provided insight into energetic aspects of how phase change from W to LP can have a very significant effect on the overall stability and 3D structure of most proteins. Based on these, a schematic energy surface of the unfolding process coupled to phase transition was obtained (Figure 4B). The latter provides a quick qualitative interpretation on the effect of phase transfer on the folding or unfolding preference of sheet motifs. We hope that our investigations not only help in better understanding the LP phase of membraneless organelles and the lipophilic interior of biomembranes but also provide quantitative information on why an inner separated hydrophobic region is required for chaperones to drive protein folding more effectively. Furthermore, the obtained results on energetic properties of the peptide backbone can also be relevant in other phenomena related to phase transition, such as the interaction between disordered membrane-active peptides and lipid bilayers.

## Data Availability

All data presented in this study are available on request from the corresponding author.

## Supplementary Materials

The following are available online at https://www.mdpi.com/article/10.3390/ijms22168595/s1.

## References

[1] Boeynaems S., Alberti S., Fawzi N.L., Mittag T., Polymenidou M., Rousseau F., Schymkowitz J., Shorter J., Wolozin B., Bosch L.V.D. (2018). Protein Phase Separation: A New Phase in Cell Biology. Trends Cell Biol..

[2] Gomes E., Shorter J. (2019). The molecular language of membraneless organelles. J. Biol. Chem..

[3] Xu Z., Horwich A.L., Sigler P.B. (1997). The crystal structure of the asymmetric GroEL–GroES–(ADP) 7 chaperonin complex. Nat. Cell Biol..

[4] Hanson P.I., Whiteheart S. (2005). AAA+ proteins: Have engine, will work. Nat. Rev. Mol. Cell Biol..

[5] Zozulia O., Korendovych I.V. (2020). Semi-Rationally Designed Short Peptides Self-Assemble and Bind Hemin to Promote Cyclopropanation. Angew. Chem..

[6] Brangwynne C.P., Eckmann C.R., Courson D.S., Rybarska A., Hoege C., Gharakhani J., Jülicher F., Hyman A.A. (2009). Germline P Granules Are Liquid Droplets That Localize by Controlled Dissolution/Condensation. Science.

[7] Hughes M.P., Sawaya M.R., Boyer D.R., Goldschmidt L., Rodriguez J.A., Cascio D., Chong L., Gonen T., Eisenberg D.S. (2018). Atomic structures of low-complexity protein segments reveal kinked β sheets that assemble networks. Science.

[8] Booth D.R., Sunde M., Bellotti V., Robinson C.V., Hutchinson W.L., Fraser P.E., Hawkins P.N., Dobson C.M., Radford S., Blake C.C.F. (1997). Instability, unfolding and aggregation of human lysozyme variants underlying amyloid fibrillogenesis. Nat. Cell Biol..

[9] Laganowsky A., Liu C., Sawaya M.R., Whitelegge J.P., Park J., Zhao M., Pensalfini A., Soriaga A.B., Landau M., Teng P.K. (2012). Atomic View of a Toxic Amyloid Small Oligomer. Science.

[10] Chuang E., Hori A., Hesketh C.D., Shorter J. (2018). Amyloid assembly and disassembly. J. Cell Sci..

[11] Feng B., Sosa R.P., Mårtensson A.K.F., Jiang K., Tong A., Dorfman K.D., Takahashi M., Lincoln P., Bustamante C.J., Westerlund F. (2019). Hydrophobic catalysis and a potential biological role of DNA unstacking induced by environment effects. Proc. Natl. Acad. Sci. USA.

[12] Starikov E.B., Nordén B. (2007). Enthalpy−Entropy Compensation: A Phantom or Something Useful?. J. Phys. Chem. B.

[13] Mason T.O., Michaels T.C.T., Levin A., Dobson C.M., Gazit E., Knowles T.P.J., Buell A.K. (2017). Thermodynamics of Polypeptide Supramolecular Assembly in the Short-Chain Limit. J. Am. Chem. Soc..

[14] Baldwin A.J., Knowles T., Tartaglia G.G., Fitzpatrick A.W., Devlin G.L., Shammas S.L., Waudby C., Mossuto M.F., Meehan S., Gras S. (2011). Metastability of Native Proteins and the Phenomenon of Amyloid Formation. J. Am. Chem. Soc..

[15] Dobson C.M. (2003). Protein folding and misfolding. Nature.

[16] Legname G., Baskakov I.V., Nguyen H.-O.B., Riesner D., Cohen F.E., DeArmond S.J., Prusiner S.B. (2004). Synthetic Mammalian Prions. Science.

[17] Wimley W.C. (2003). The versatile β-barrel membrane protein. Curr. Opin. Struct. Biol..

[18] Perczel A., Hudáky A.P., Pálfi V.K. (2007). Dead-End Street of Protein Folding: Thermodynamic Rationale of Amyloid Fibril Formation. J. Am. Chem. Soc..

[19] Navizet L., Cailliez F., Lavery R. (2004). Probing Protein Mechanics: Residue-Level Properties and Their Use in Defining Domains. Biophys. J..

[20] Dougan L., Feng G., Lu H., Fernandez J.M. (2008). Solvent molecules bridge the mechanical unfolding transition state of a protein. Proc. Natl. Acad. Sci. USA.

[21] Morozov A.V., Kortemme T. (2005). Potential Functions for Hydrogen Bonds in Protein Structure Prediction and Design. Advances in Protein Chemistry.

[22] Paci E., Karplus M. (1999). Forced unfolding of fibronectin type 3 modules: An analysis by biased molecular dynamics simulations. J. Mol. Biol..

[23] Beke-Somfai T., Perczel A. (2010). Zipper-Like Unfolding of β-Sheets Accessed by Pioneer Water Molecules: Atomic Resolution of Forced Unfold Reveals Different Mechanisms for Parallel and Antiparallel Motifs. J. Phys. Chem. Lett..

[24] Morozov A.V., Kortemme T., Tsemekhman K., Baker D. (2004). Close agreement between the orientation dependence of hydrogen bonds observed in protein structures and quantum mechanical calculations. Proc. Natl. Acad. Sci. USA.

[25] Pohl G., Beke T., Borbély J., Perczel A. (2006). Prediction of Folding Preference of 10 kDa Silk-like Proteins Using a Lego Approach and ab Initio Calculations. J. Am. Chem. Soc..

[26] Kortelainen M., Suhonen A., Hamza A., Papai I., Nauha E., Yliniemelä-Sipari S., Nissinen M., Pihko P.M. (2015). Folding Patterns in a Family of Oligoamide Foldamers. Chem. A Eur. J..

[27] Johansson J.R., Hermansson E., Nordén B., Kann N., Beke-Somfai T. (2014). δ-Peptides from RuAAC-Derived 1,5-Disubstituted Triazole Units. Eur. J. Org. Chem..

[28] Himo F. (2017). Recent Trends in Quantum Chemical Modeling of Enzymatic Reactions. J. Am. Chem. Soc..

[29] Sharma D., Perišić O., Peng Q., Cao Y., Lam C., Lu H., Li H. (2007). Single-molecule force spectroscopy reveals a mechanically stable protein fold and the rational tuning of its mechanical stability. Proc. Natl. Acad. Sci. USA.

[30] Banerjee P.R., Deniz A.A. (2013). Shedding Light on Protein Folding Landscapes by Single-molecule Fluorescence. Chem. Soc. Rev..

[31] Narimoto T., Sakurai K., Okamoto A., Chatani E., Hoshino M., Hasegawa K., Naiki H., Goto Y. (2004). Conformational stability of amyloid fibrils of β2 -microglobulin probed by guanidine-hydrochloride-induced unfolding. FEBS Lett..

[32] Mardirossian N., Head-Gordon M. (2017). Thirty years of density functional theory in computational chemistry: An overview and extensive assessment of 200 density functionals. Mol. Phys..

[33] Papp D., Rovó P., Jákli I., Császár A.G., Perczel A. (2017). Four faces of the interaction between ions and aromatic rings. J. Comput. Chem..

[34] Shu C., Jiang Z., Biczysko M. (2020). Toward accurate prediction of amino acid derivatives structure and energetics from DFT: Glycine conformers and their interconversions. J. Mol. Model..

[35] Alabugin I.V., Manoharan M., Peabody S., Weinhold F. (2003). Electronic Basis of Improper Hydrogen Bonding: A Subtle Balance of Hyperconjugation and Rehybridization. J. Am. Chem. Soc..

[36] Rabuck A.D., Scuseria G.E. (2000). Performance of recently developed kinetic energy density functionals for the calculation of hydrogen binding strengths and hydrogen-bonded structures. Theor. Chem. Acc..

[37] Boese A.D. (2015). Density Functional Theory and Hydrogen Bonds: Are We There Yet?. ChemPhysChem.

[38] Luccarelli J., Paton R.S. (2018). Hydrogen-Bond-Dependent Conformational Switching: A Computational Challenge from Experimental Thermochemistry. J. Org. Chem..

[39] Pepin R., Laszlo K.J., Marek A., Peng B., Bush M.F., Lavanant H., Afonso C., Tureček F. (2016). Toward a Rational Design of Highly Folded Peptide Cation Conformations. 3D Gas-Phase Ion Structures and Ion Mobility Characterization. J. Am. Soc. Mass Spectrom..

[40] Perczel A., Gáspári Z., Csizmadia I.G. (2005). Structure and stability of β-pleated sheets. J. Comput. Chem..

[41] Perczel A., Angyan J.G., Kajtar M., Viviani W., Rivail J.L., Marcoccia J.F., Csizmadia I.G. (1991). Peptide models. 1. Topology of selected peptide conformational potential energy surfaces (glycine and alanine derivatives). J. Am. Chem. Soc..

[42] Beke T., Czajlik A., Csizmadia I.G., Perczel A. (2006). Determining suitablelego-structures to estimate stability of larger peptide nanostructures using computational methods. Phys. Biol..

[43] Sastre S., Casasnovas R., Muñoz F., Frau J. (2016). Isodesmic reaction for accurate theoretical pK a calculations of amino acids and peptides. Phys. Chem. Chem. Phys..

[44] Wimley W.C., White S. (1996). Experimentally determined hydrophobicity scale for proteins at membrane interfaces. Nat. Struct. Mol. Biol..

[45] Wimley W.C., Creamer T.P., White S. (1996). Solvation Energies of Amino Acid Side Chains and Backbone in a Family of Host–Guest Pentapeptides. Biochemistry.

[46] Baker T.A., Sauer R.T. (2012). ClpXP, an ATP-powered unfolding and protein-degradation machine. Biochim. Biophys. Acta (BBA) Mol. Cell Res..

[47] Hill K., Model K., Ryan M.T., Dietmeier K., Martin F., Wagner R., Pfanner N. (1998). Tom40 forms the hydrophilic channel of the mitochondrial import pore for preproteins. Nature.

[48] Fei X., Bell T.A., Jenni S., Stinson B.M., Baker T.A., Harrison S.C., Sauer R.T. (2020). Structures of the ATP-fueled ClpXP proteolytic machine bound to protein substrate. eLife.

[49] Buell A.K., Dhulesia A., Mossuto M.F., Cremades N., Kumita J.R., Dumoulin M., Welland M.E., Knowles T., Salvatella X., Dobson C.M. (2011). Population of Nonnative States of Lysozyme Variants Drives Amyloid Fibril Formation. J. Am. Chem. Soc..

[50] Beke T., Csizmadia I.G., Perczel A. (2006). Theoretical Study on Tertiary Structural Elements of β-peptides: Nanotubes Formed from Parallel-Sheet-Derived Assemblies of β-Peptides. J. Am. Chem. Soc..

[51] And R.F.W.B., Bayles D. (2000). Properties of Atoms in Molecules: Group Additivity. J. Phys. Chem. A.

[52] Cances E., Mennucci B., Tomasi J. (1997). A new integral equation formalism for the polarizable continuum model: Theoretical background and applications to isotropic and anisotropic dielectrics. J. Chem. Phys..

[53] Stewart J.J.P. (2012). Optimization of parameters for semiempirical methods VI: More modifications to the NDDO approximations and re-optimization of parameters. J. Mol. Model..

[54] Řezáč J., Fanfrlik J., Salahub D., Hobza P. (2009). Semiempirical Quantum Chemical PM6 Method Augmented by Dispersion and H-Bonding Correction Terms Reliably Describes Various Types of Noncovalent Complexes. J. Chem. Theory Comput..

