# Supplementary material for: Structural Water Stabilizes Protein Motifs in Liquid Protein Phase: The Folding Mechanism of Short β-Sheets Coupled to Phase Transition
Source: Int J Mol Sci. 2021 Aug 10;22(16):8595. doi: 10.3390/ijms22168595 (PMC8395295; doi:10.3390/ijms22168595)
Supplement: Supplementary file 1 [file ijms-22-08595-s001.zip › Supplementary_ijms_1285568_proof_corrected.pdf]

Structural Water Stabilizes Protein Motifs in Liquid Protein Phase: The Folding Mechanism of Short β-Sheets Coupled to Phase Transition

Dóra Papp[1,2], Imola Csilla Szigyártó[3], Bengt Nordén[4], András Perczel[1], Tamás Beke-Somfai[3*]

[1] Laboratory of Structural Chemistry and Biology, MTA ELTE Protein Modelling Research Group, Institute of Chemistry, Eötvös Loránd University, Pázmány Péter Sétány 1/A, H-1117 Budapest, Hungary; perczel@chem.elte.hu (A.P.)

[2] MTA-SZTE Lendület Computational Reaction Dynamics Research Group, Interdisciplinary Excellence Centre and Department of Physical Chemistry and Materials Science, Institute of Chemistry, University of Szeged, Rerrich Béla tér 1, Szeged H-6720, Hungary; dorapapp@chem.u-szeged.hu (D.P.)

[3] Biomolecular Self-Assembly Research Group, Institute of Materials and Environmental Chemistry, Research Centre for Natural Sciences, Magyar tudósok körútja 2., Budapest H-1117, Hungary; szigyarto.imola.csilla@ttk.hu (I.Cs.Sz.); beke-somfai.tamas@ttk.hu (T.B.-S.)

[4] Department of Chemical and Biological Engineering, Physical Chemistry, Chalmers University of Technology, SE-412 96 Göteborg, Sweden; norden@chalmers.se (B.N.)

* Correspondence: beke-somfai.tamas@ttk.hu

This PDF file includes:

Supplementary text
Figures S1 to S5
Legend for Video SI_3Turn_9w_close
SI References

Other supplementary materials for this manuscript include the following:

Movie SI_3Turn_9w_close

Supplementary Information Text

Methods

Energetic effect of phase separation on folding

To estimate the effect of environmental changes isodesmic equations were employed [1–4]. Accordingly, to model the W to LP at different stages of the unfolding event were split into two parts in such a way that the already unfolded part of the β-sheet was simulated in apolar media imitating the LP phase while the rest of the structure was considered to be in W. Energies of the total system was obtained as follows

$$\text{X-x}_{\text{water}} + \text{y-Y}_{\text{protein}} \rightarrow \text{X-Y}_{\text{motif translocating}} + \text{x-y}_{\text{byproduct}} \qquad \textbf{(Eq. S1)}$$

$$\Delta E_{\text{ID}} = \{ E[\text{X-x}] + E[\text{y-Y}]\} - \{E[\text{X-Y}] + E[\text{x-y}]\} \qquad \textbf{(Eq. S2)},$$

where X and Y are the two parts of the partially unfolded motif translocating into a protein environment, X is still in water, whereas Y is already in LP (**Figure 4**). For terminals, *y* and *x* represent a formyl and an amino group, respectively and thus, the byproduct of the isodesmic reaction, *x-y*, is a formamide. Note, that formamide molecule was also optimized at the same level of theory as X-x and Y-y. In the case when there are no close interactions between the X and Y fragments ΔE_{ID} is zero and thus *E*[X-Y] can be obtained (**Eq. S3**)

$$E[\text{X-Y}] = E[\text{X-x}] + E[\text{y-Y}] - E[\text{x-y}]\} \quad \textbf{(Eq. S3)}$$

Similarly, the isodesmic relative energy (ΔE_{ID}) of the reverse process can be obtained based on (**Eq. S2**), but considering the motifs in a reverse manner.

Figures

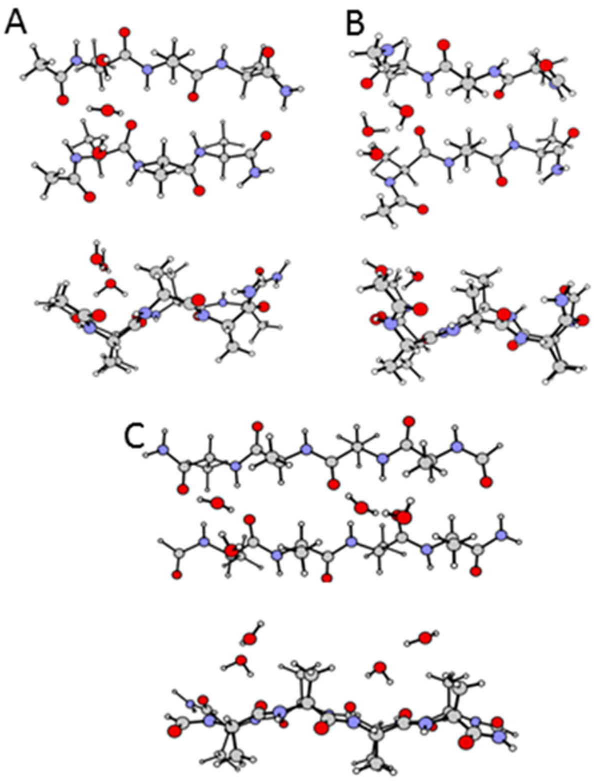

Figure S1. Top and side view of the β-sheet models used in this study. **A**: The parallel strand model with 3 water molecules (P3) coordinated on the N-terminal peptide groups. **B**: The antiparallel model with 3 water molecules (AP3). **C**: The antiparallel model with 4 water molecules used for shear unfolding (S4).

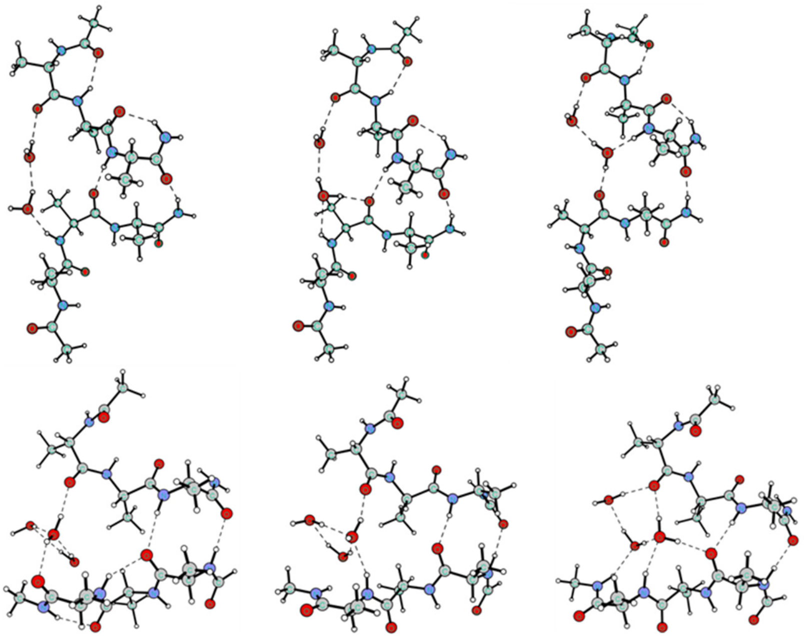

Figure S2. Structural water molecules moving in between two β-strands comprising β-sheet motifs during a stepwise unfolding event. Displayed structures for lateral unfolding of the *P2* (UPPER PANELS) and *AP3* models (LOWER PANELS). For *P2* scanning distances are 16 Å, 17 Å and 18 Å, left to right. For *AP3*, are 10Å, 11Å, and 12Å, left to right [5].

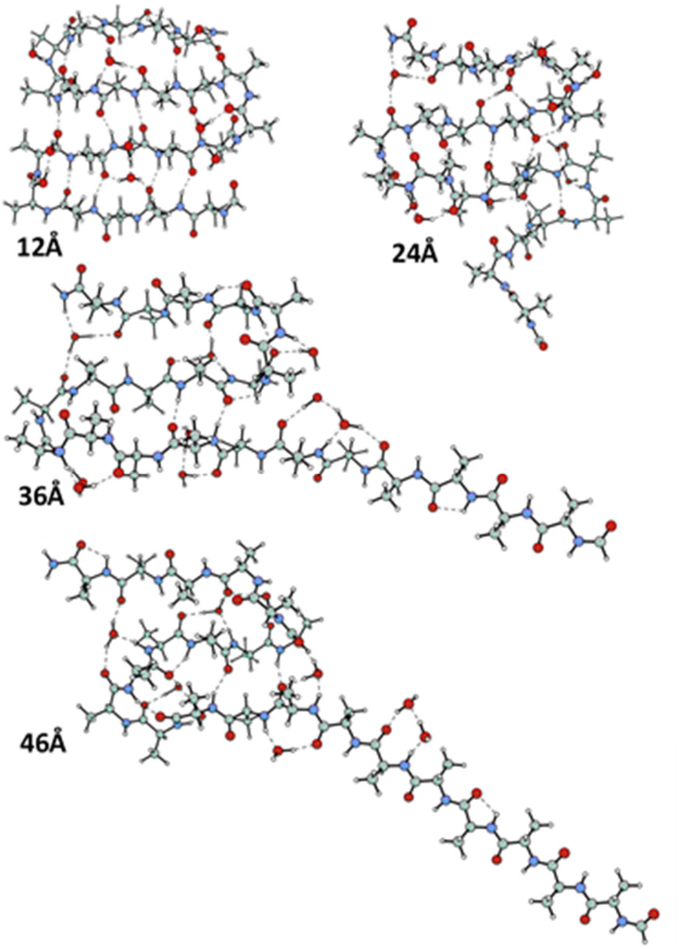

Figure S3. Snapshots along the stepwise unfold of the 22 amino acid long 3Turn model. The initial stage consists of the lateral unfolding at the *N*-terminal residues. As this β-strand is unfolded, a sheared unfold takes place, followed by a sudden rearrangement within the original β-sheet motif resulting in a shorter β-sheet, but with the same number of peptide strands. Displayed values mark the distances between the two terminal peptide bonds.

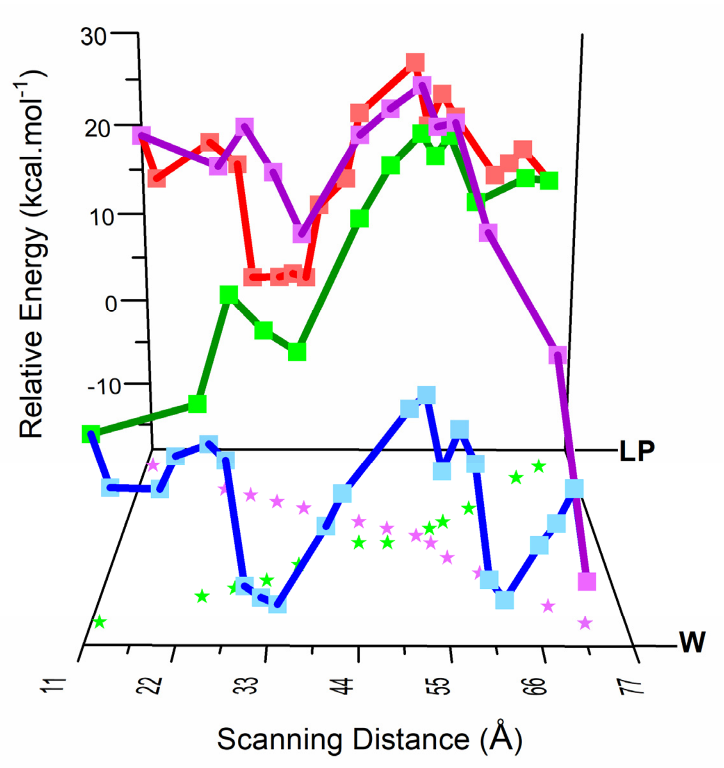

Figure S4. Conversion between W and LP phase energy landscapes during unfolding. By pulling the elongated part in the bulk protein environment, the system minimalizes the requested energy to convert from the bulk water (favorable) to the bulk protein (less favorable). LP environment shown as red, W as blue.

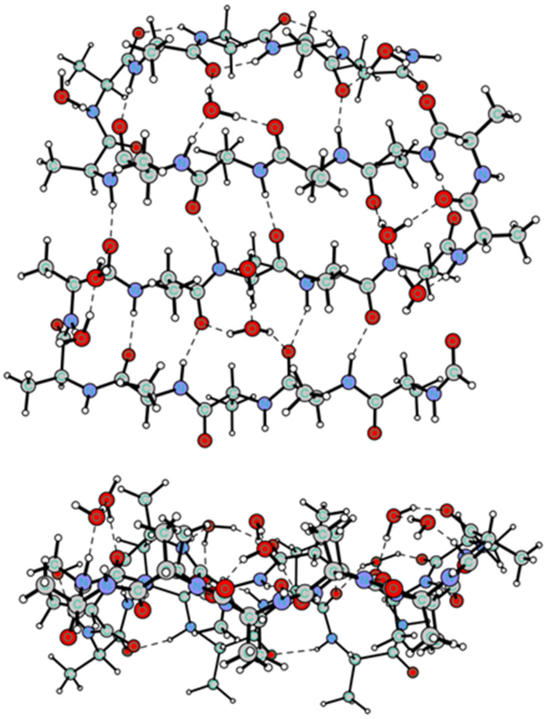

Figure S5. Top and side view of model 3Turn, with 9 water molecules coordinated on the protein motif representing the protein external surface – structural water interaction

Video SI_3Turn_9w_close (separate file). The stepwise scan on the unfolding of model 3Turn, with a setup that enables following the break-up of H-bonds and the melting of sheet strands

References

(1) Pohl, G.; Beke, T.; Borbély, J.; Perczel, A. Prediction of Folding Preference of 10 KDa Silk-like Proteins Using a Lego Approach and Ab Initio Calculations. *J. Am. Chem. Soc.* **2006**, *128* (45), 14548–14559. https://doi.org/10.1021/ja063933p.

(2) Beke, T.; Czajlik, A.; Csizmadia, I. G.; Perczel, A. Determining Suitablelego-Structures to Estimate Stability of Larger Peptide Nanostructures Using Computational Methods. *Phys. Biol.* **2006**, *3* (1), S26–S39. https://doi.org/10.1088/1478-3975/3/1/S04.

(3) Beke, T.; Csizmadia, I. G.; Perczel, A. Theoretical Study on Tertiary Structural Elements of β-Peptides: Nanotubes Formed from Parallel-Sheet-Derived Assemblies of β-Peptides. *J. Am. Chem. Soc.* **2006**, *128* (15), 5158–5167. https://doi.org/10.1021/ja0585127.

(4) Bader, R. F. W.; Bayles, D. Properties of Atoms in Molecules: Group Additivity. *J. Phys. Chem. A* **2000**, *104* (23), 5579–5589. https://doi.org/10.1021/jp9943631.

(5) Beke-Somfai, T.; Perczel, A. Zipper-Like Unfolding of β-Sheets Accessed by Pioneer Water Molecules: Atomic Resolution of Forced Unfold Reveals Different Mechanisms for Parallel and Antiparallel Motifs. *J. Phys. Chem. Lett.* **2010**, *1* (9), 1341–1345. https://doi.org/10.1021/jz100311n.
